# A Structural Split in the Human Genome

**DOI:** 10.1371/journal.pone.0000603

**Published:** 2007-07-11

**Authors:** Clara S.M. Tang, Richard J. Epstein

**Affiliations:** Laboratory of Computational Oncology, Department of Medicine, The University of Hong Kong, Pokfulam, Hong Kong, Hong Kong; Genome Institute of Singapore, Singapore

## Abstract

**Background:**

Promoter-associated CpG islands (PCIs) mediate methylation-dependent gene silencing, yet tend to co-locate to transcriptionally active genes. To address this paradox, we used data mining to assess the behavior of PCI-positive (PCI+) genes in the human genome.

**Results:**

PCI+ genes exhibit a bimodal distribution: (1) a ‘housekeeping-like’ subset characterized by higher GC content and lower intron length/number, and (2) a ‘pseudogene paralog’ subset characterized by lower GC content and higher intron length/number (*p*<0.001). These subsets are functionally distinguishable, with the former gene group characterized by higher expression levels and lower evolutionary rate (*p*<0.001). PCI-negative (PCI-) genes exhibit higher evolutionary rate and narrower expression breadth than PCI+ genes (*p*<0.001), consistent with more frequent tissue-specific inactivation.

**Conclusions:**

Adaptive evolution of the human genome appears driven in part by declining transcription of a subset of PCI+ genes, predisposing to both CpG→TpA mutation and intron insertion. We propose a model of evolving biological complexity in which environmentally-selected gains or losses of PCI methylation respectively favor positive or negative selection, thus polarizing PCI+ gene structures around a genomic core of ancestral PCI- genes.

## Introduction

Evolution of biological complexity involves an environmentally-regulated balance between genetic conservation and variation [1]–[6]. Phylogenetic leaps favoring speciation of higher organisms include the evolution of introns and DNA methylation [7], [8]. A more recent innovation is that of promoter-associated CpG islands (PCIs) [9] which, when methylated, mediate transcriptional repression and/or chromatin condensation [10]. About 60% of human genes contain PCIs [9], most of which are unmethylated [11], [12]. Such PCIs are more common in widely-expressed (housekeeping) genes [13], supporting the view that non-methylated PCIs actively maintain gene transcription [14], [15].

In addition to the functional effects of PCI methylation on transcription, methylcytosine residues in coding regions may undergo oxidative deamination to thymine, with such events being quantifiable as an excess of CG→TA transitional mutations[16]. This interaction between methylation-dependent *trans*-repression and mutation drives adaptive evolution [17]. The latter observation is in turn consistent with the finding that methylation of CpG-rich regions facilitates transposition events [18].

A single interpretation of PCI behavior in transcription and evolution thus remains elusive [19], [20], suggesting a more ‘kaleidoscopic’ model in which PCI significance oscillates with methylation status. Relevant to this, we showed in earlier work that CpG dinucleotide retention in coding sequences correlates with codon essentiality [21], [22], whereas both gene methylation and intron length accelerate the evolution of less well repaired downstream intragenic sequences [23]. These results suggest a dynamic pro-evolutionary balance between negative selection for retention of CpG dinucleotides in transcriptionally active genes, and adaptive evolution for methylation-dependent CpG mutation in transcriptionally inactive genes. The present study was designed to test this hypothesis by comparing PCI-containing (PCI+) and PCI-deficient (PCI-) genes, and has identified in the process two structurally and functionally distinct PCI+ gene subsets.

## Results

### The GC nucleotide content of PCI-containing human genes exhibits a biphasic distribution

**Figure 1:**
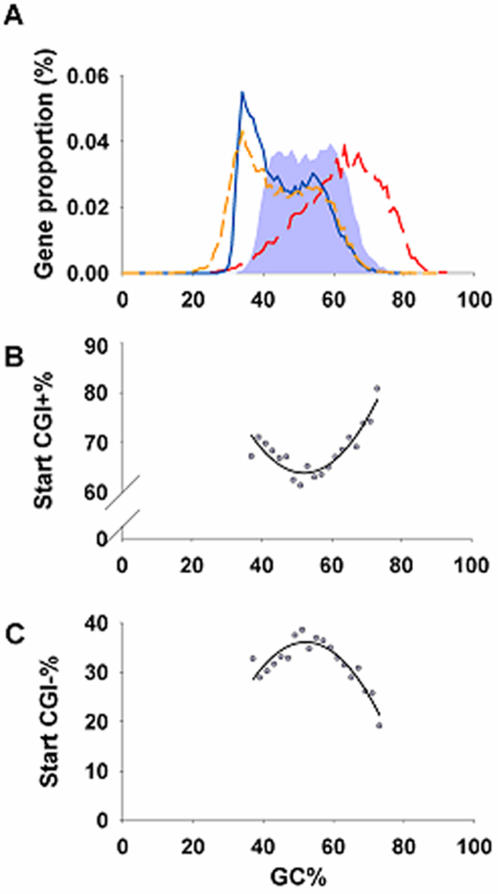
Biphasic GC/AT distribution of PCI+ genes. A, Distribution of GC content among different regions of genes (coding region, grey fill; intronic, black solid; 5¢ untranslated region, dark grey, long dash; 3¢ untranslated region, grey, short dash). B&C, Proportion of genes among different GC groups; B, with ‘start’ CpG islands (CGI+). C, without ‘start’ CpG islands (CGI-).

We first compared the genomic architecture of human genes with that of lower organisms. This confirmed that the human genome is characterized not only by higher CpG island frequency and greater intron number and length [9], but also by a higher coefficient of variation and more negative kurtosis of its nucleotide content (G+C; Table 1). This finding raised the possibility that the notably broad GC range of the human DNA distribution (Figure 1A) relates in some manner to the pro-evolutionary events of CpG island acquisition and/or intron insertion. To address this possibility, we next distinguished ‘start CpG islands’ from downstream CpG islands (see Methods). As shown in Figure 1B, the frequency distribution of start CpG islands exhibits two maxima based on GC content, mirroring the single peak of genes lacking start CpG islands (Figure 1C). A similar pattern is evident when the search is restricted to promoter-associated CpG islands (PCIs), implying that the distribution of PCI-negative genes peaks at a single intermediate GC content. Control comparisons of genes with unidirectional *vs.* bidirectional promoters revealed no significant differences in intron number, evolutionary rate or expression level (**Supplement S1**).

**Table 1:** Cross-species comparison of coding GC content (c.v., kurtosis, and skewness), proportion of genes with promoter-associated CpG islands (PCI), number of exons, and intron length.

	*C. elegans*	*D. melanogaster*	*T. rubripes*	*M. musculus*	*H. sapiens*
**Mean coding GC%**	40.64	53.95	53.80	51.44	52.78
**Coefficient of variation**	0.12	0.09	0.09	0.14	0.16
**Excess kurtosis**	0.19	2.25	5.64	1.26	−0.83
**Skewness**	0.39	−0.92	−0.75	−0.51	0.12
**Proportion of PCI+ genes (%)**	0.33	13.91	25.55	59.81	67.00
**Mean number of exons**	6.44	4.68	4.80	9.09	10.52
**Intron length (kb)**	1.60	5.29	4.10	45.69	58.48

**Figure 2:**
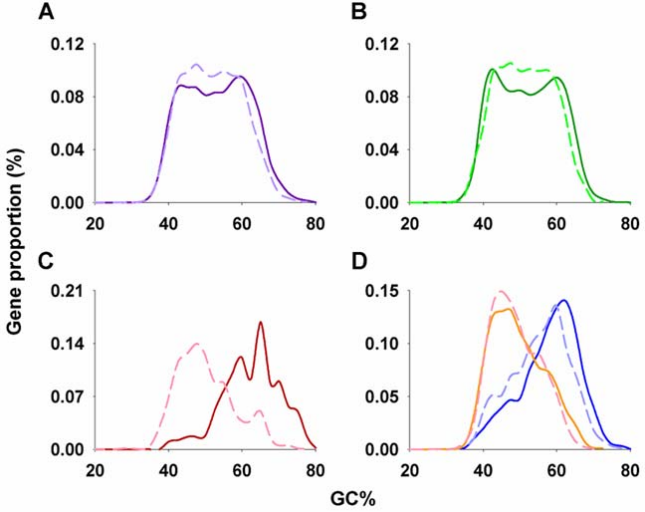
Distribution of GC content of promoter CpG island overlapping genes (PCI+, solid line) and non-promoter CpG island overlapping genes (PCI-, dash line). A, All genes; B, Genes with medium total intron size (10–50 kb); C, intronless genes; and D, genes with short total intron size (<10 kb, blue) and long intron size (>50 kb, orange).

Figure 2 analyzes in more detail the GC contents of PCI+ and PCI- genes. These patterns appear similar in the whole genome (Fig. 2A) and in genes with intermediate intron lengths (Fig. 2B). However, PCI+ genes lacking introns or containing only short overall intron lengths are characterized by high GC content (Fig. 2C, solid line), whereas PCI+ genes containing long introns are characterized by low GC content (Fig. 2D).

**Figure 3:**
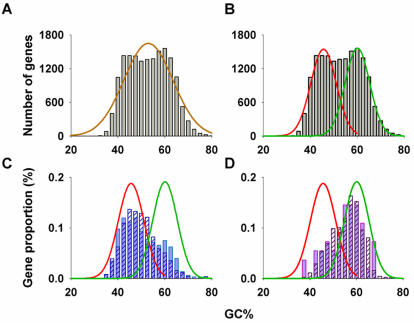
Distribution of coding GC% of RefGenes with PCIs. Unimodal (A) and bimodal distribution curves (B) were fitted to coding GC% distribution. C, Coding GC% of paralogs of processed pseudogenes with (light blue filled) and without PCIs (open blue stroke) shift towards lower GC content. D, Coding GC% of housekeeping genes with (purple fill) and without PCIs (purple stroke) shift towards higher GC. See Methods for subset derivation.

The PCI+ gene nucleotide distribution was further examined using a computational curve comparison, confirming a better fit for a bimodal distribution (Figure 3B; peaks of 46.09+/−5.19, and 60.27+/−5.19) than for a unimodal distribution (Figure 3A; mean 53.13+/−8.78; *p*<0.001). These patterns resemble those of ‘pseudogene paralog’ and ‘housekeeping-like’ gene clusters (Figs. 3C and 3D respectively), consistent with the possibility that gene expression contributes to evolutionary changes in GC content among PCI+ genes (see **Supplement S2** for validation of the foregoing functional definitions).

### High-GC and low-GC PCI+ gene clusters are distinguishable in terms of intron content, evolutionary rate, and expression

**Figure 4:**
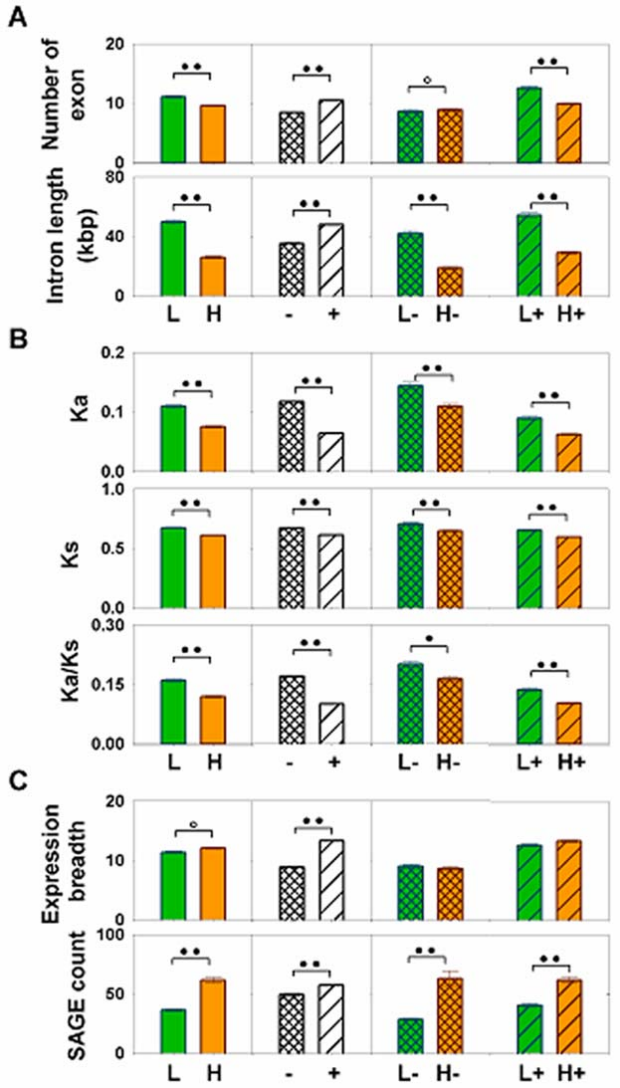
Quantitative comparison of gene subsets characterized in terms of PCI status, intron length/number, GC content, evolutionary rate, expression breadth and expression level. For expression levels, trimmed means (5%) with standard errors were used, with promoter non-overlapping (-, cross) and overlapping CpG islands (+, stroke) of different GC content. (Low, L, GC<40%, black; high, H, GC>65%, grey). P-value of nonparametric KS test is shown in circles (double dark, <0.001; single dark, <0.01; open, <0.05). A, Intron length and number; B, Evolutionary rate (Ka/Ks); C, Expression breadth and level.

The relationship between the structural variables of PCI status, GC content, intron number, and total intron length was then examined. As shown in Figure 4A, significant increases in both intron number and intron length characterise both AT-rich and PCI+ gene subsets. This is also true for AT-rich PCI+ genes; however, for AT-rich PCI- genes, the increase in intron number is only borderline, even though intron length remains a highly significant differential. These findings raise the hypothesis that PCIs play a causal role in intron insertion, perhaps via chromatin-related effects, whereas the correlation between low GC content and total intron length could reflect other factors (e.g., transcription, repair, etc.).

Like intron number and length, evolutionary rate–quantified here by the Ka/Ks ratio–is increased in AT-rich compared to GC-rich genes; in contrast to the foregoing intronic correlations, however, PCI+ genes exhibit a lower evolutionary rate that is synergistic with high GC content (Fig. 4B). This implies that PCI+ genes feature higher transcription/repair or greater negative selection pressure than PCI- genes–which remains consistent with the hypothesis that PCIs favor intron insertion via a separate chromatin effect.

Figure 4C supports this latter possibility, showing as it does that gene expression breadth is greatly increased in PCI+ (vs. PCI-) genes, but not in GC-rich (vs. AT-rich) genes. In contrast, average expression levels are higher in GC-rich than in AT-rich genes, particularly in PCI- gene subsets. Since changes in expression breadth tend to be mediated by tissue-specific differences in chromatin organization, these data support earlier analyses concluding that PCI-dependent effects on chromatin structure do not closely parallel methylation-dependent effects on transcription [24]. In spite of the over-representation of promoter CpG islands in divergent promoter genes, which is thought to regulate coordinated gene expression, this group of genes exhibited PCI+ structural characterisitics similar to those of unidirectional ones (**Supplement S1, Figure S1, Table S1, Table S2**) .

**Figure 5:**
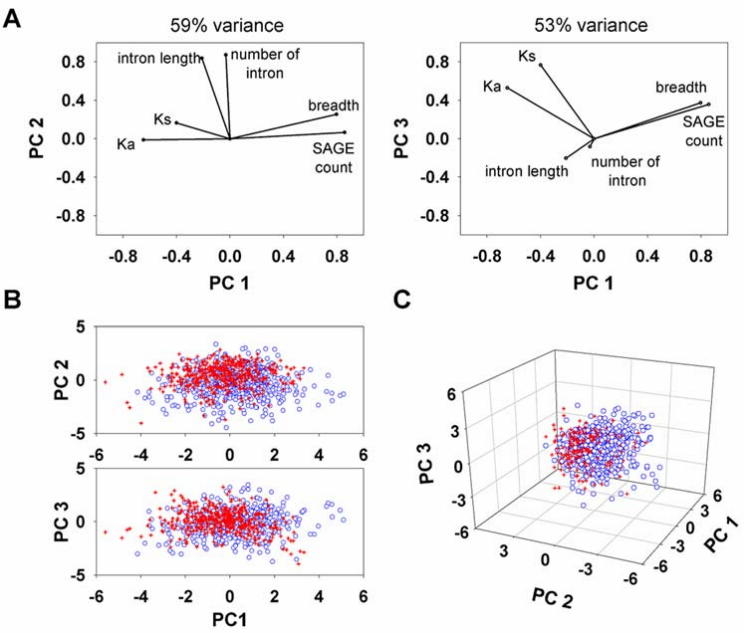
Principal component analysis (PCA). A, PCA analysis using six variables at either 53% (left) or 59% (right) variance. B, Two-dimensional dot plots confirming the existence of distinct PCI+ gene subsets (red cross, low GC; blue circle, high GC) based on intron size/number, transcription, and evolutionary rate. C, Three-dimensional dot plot of the GC-rich (blue) and GC-poor (red) PCI+ gene clusters.

**Figure 6:**
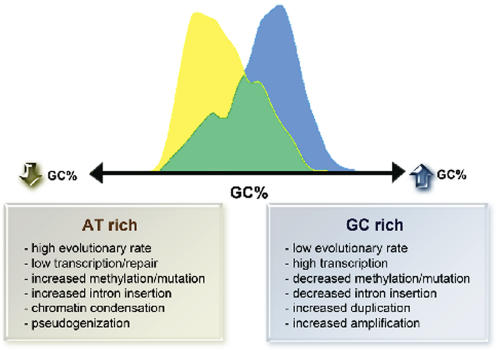
Model of human genomic evolution, proposing the development of a structural split in GC content based on the association of methylation-dependent mutation with transcriptional repression, chromatin condensation, reduced repair and intron insertion.

The existence of gene subgroups structurally separable on the basis of PCI status, GC content, intron length and intron number is confirmed by principal component analysis (Fig. 5A–C). This suggests a model of genetic evolution in which the speed of evolution towards greater biological complexity is maximized by concurrent positive and negative selection forces acting differentially on the genome in response to environmental changes, leading in turn to a selectable balance between gene expression and structural fidelity (Fig. 6).

## Discussion

There are three central findings of this study. First, there exists an AT-rich ‘second peak’ of PCI+ genes which, when compared with the GC-rich peak of housekeeping-like PCI+ genes, is characterized by lower transcriptional activity, higher intron number and length, and higher evolutionary rate. We propose that the AT-rich subset has arisen from the GC-rich subset of PCI+ genes via progressive loss of negative selection pressure, accompanied by progressive PCI methylation.

Second, PCI- genes have a higher evolutionary rate and lower expression breadth than PCI+ genes. This suggests either that (i) only widely-transcribed (housekeeping-like) genes are under selection pressure to acquire or retain PCIs, and/or (ii) PCI loss represents a separate pathway towards pseudogenization (e.g., mediated by heterochromatinization) for less-transcribed tissue-specific genes. Since PCI frequency increases with biological complexity (Table 1), we submit that the majority of PCI- genes represent ancestral genes, for which an increase in tissue-specific negative selection pressure could favor PCI acquisition (e.g., via transposition events).

Third, although total intron length correlates best with AT-richness, intron number relates more directly to PCI positivity (Fig 4A). Given both the CG→TA mutability of methylated CpG sites, and the association of PCI methylation with chromatin condensation, this finding suggests that intron extension could occur via an autocatalytic mechanism [25] associated with reduced repair, whereas intron insertion per se is dependent upon PCI-dependent chromatin alterations. Since the stronger correlation between PCI+ genes (>GC-rich genes) and expression breadth (>expression level; Fig. 4C) implicates PCI-dependent chromatin activation–i.e., even in the presence of transcriptional repression and impaired repair of methylation-dependent mutation–as a causal mechanism, PCI-dependent chromatin effects could likewise mediate the greater intron length and number characteristic of mammalian genomes. This hypothesis is consistent with the pro-evolutionary nature of mutations eluding mismatch repair [26] as well as with the presence of ‘transition regions’ between euchromatin and heterochromatin [27], [28].

Hence, our findings suggest a paradigm of PCIs not only as transcriptional regulators, but also as evolutionary accelerators that can facilitate intron insertion–perhaps via modulation of chromatin structure [29]–under environmental circumstances in which transcriptional inhibition does not compromise fitness. We agree with others [30] that the positive correlations of PCIs with both transcription level/breadth and with intron number/length do not support a simplistic “selection for time economy” model of intron evolution. Moreover, we stress that a full molecular elucidation of the present hypothesis must be awaited.

In conclusion, the present study has identified two subsets of PCI+ genes within the human genome. We propose that the opposing consequences of differentially methylated PCIs on transcription and chromatin accelerate adaptive evolution towards biological complexity. As such, this study supports the view that the reversible methylation-dependent mechanism of structural switching between these functional extremes constitutes nothing less than an evolutionary masterstroke of natural selection.

## Materials and Methods

### Sequence data and annotations

We retrieved the genomic sequences of five species–human (*Homo sapiens*), mouse (*Mus musculus),* Fugu (*Takifugu rubripes),* fruit fly (*Drosophila melanogaster)* and worm (*Caenorhabditis elegans)*–from the University of California, Santa Cruz (UCSC) Table Browser (http://genome.ucsc.edu/) (Karolchik, et al., 2004). Genome assemblies of hg18 (NCBI build 36.1, March 2006), mm6 (NCBI Build 34, March 2005), fr1 (JGI v3.0 August 2002), dm2 (BDGP Release 4, April 2004) and ce2 (WormBase WS120, March 2004), respectively, were used. Since the transcription start site does not shift much according to DBTSS annotation (see **Supplement S3, Figure S3**), sequence analyses of all species were carried out using RefSeq genes, with the exception of Fugu for which no RefSeq dataset is available. and for which the Ensembl gene dataset was used instead.

To prevent interspersed repeats like *Alu* sequences from creating bias in nucleotide composition, RepeatMask sequences were used. Genes not commencing with ATG codons, or not terminating with canonical stop codons, were excluded in order to obtain the most reliable and homogeneous set of complete coding genes. When several genes contained identical exonic sequences, only the one with longest genomic length was retained.

### Determination of CpG island overlapping transcription start site

To identify CpG islands overlapping with promoter region, CpG islands annotation (cpgIslandExt) was downloaded from UCSC Genome Bioinformatics site, listing the physical position of CpG islands determined by the database. The position information was then mapped with RefGene annotation to isolate the RefGenes with start CGIs or promoter CGIs (PCI) whose transcriptional start site or promoter (2kb upstream and 500bp downstream) overlapped with the pre-determined CpG islands respectively.

### Housekeeping genes and paralogs of pseudogenes

Analysis of housekeeping genes was carried out using a previously defined set (Eisenberg and Levanon, 2003) containing 502 housekeeping genes. Non-overlapping set of processed pseudogenes was obtained from (http://www.pseudogene.org, Karro *et.al.*, 2006) and the RefGenes corresponding to the same proteins were mapped from the annotation, resulting in 1220 pseudogene paralogs (**Supplement S2, Figure S2**).

### Bimodal Distribution of GC content

The distribution of coding GC% was best-fitted using the NOCOM program (http://www.genemapping.cn/nocom.htm) based on a counting (EM) algorithm. Under no transformation (exponent = 1), mean, the standard deviation and proportion of each population was estimated. To test for bimodality, the bimodal distribution model was compared against the unimodal one using the statistics G^2^ = 2{} which has an asymptotic χ^2^ distribution with degrees of freedom approximate to 2 (d.f. = 2), where ln(L–_1_) and ln(L_0_) are maximum log likelihood for a bimodal and unimodal distribution respectively.

### Gene expression data

The SAGEmap (Nov 2005, ftp://ftp.ncbi.nlm.nih.gov/pub/sage) of NCBI was used for quantitative evaluation of gene expression. SAGE libraries were grouped according to 26 tissue types including brain, blood, bone, bone marrow, cervix, cartilage, colon, eye, heart, kidney, liver, lung, lymph node, mammary gland, muscle, ovary, pancreas, peripheral nervous system, placenta, prostate, skin, stem cell, stomach, thyroid, vascular and esophagus. Reliable tag-to-gene mapping of NlaIII SAGE tags to UniGene clusters was obtained from SAGEmap, and each cluster was represented by the longest RefSeq gene. Ambiguous tags mapping to more than one RefSeq gene were excluded. If a tag had been counted once only in one tissue, it was regarded as likely due to sequencing error and was thus discounted. SAGE tags of each RefGene were counted for each tissue type and normalized to counts per million. The normalized counts of each tissue were averaged across all tissue types for fair comparison between organs with different mean expression level.

### Evolutionary rate determination

Homologue data in XML format was obtained from NCBI HomoloGene database (ftp://ftp.ncbi.nih.gov/pub/HomoloGene/). Orthologous gene pairs between human and mouse, together with their synonymous substitution, non-synonymous substitution rate (Ka) and their ratio (Ka/Ks) were isolated. Genes with Ka>1.5 and Ks>3 in any quartile were discarded due to high estimation error.

### Principal component analysis (PCA)

In order to visibly corroborate the association of bimodal distribution with structural variations, we applied principal component analysis using XSTAT to gene subgroups of low and high GC content with intron size, intron number, evolutionary rate, expression breadth and level as variables. The analysis was performed using the correlation matrix, and the observations were visualized with the first three principal components in two and three dimensions.

## Supporting Information

Supplement S1 Supplement 1.
(0.03 MB DOC)

Supplement S2 Supplement 2.
(0.03 MB DOC)

Supplement S3 Supplement 3.
(0.03 MB DOC)

Table S1 Distribution of genes with divergent promoters, as characterised by the distance between transcriptional start sites.
(0.02 MB DOC)

Table S2 Proportion of divergent promoters with CpG islands among genes with different distances between transcription start sites vs. all genes.
(0.02 MB DOC)

Figure S1 Structural characterization (Upper panel : GC content, total intron length and number of intron(s), expression breath; Lower panel : Ka, Ks, Ka/Ks and expression level) of three groups of divergent promoters, overlapping or <0.3kb (pink); 0.3-1kb (red); 1-10kb (dark red) against all genes (light blue; shaded).
(0.85 MB TIF)

Figure S2 Median and quartile expression level and breadth of housekeeping genes and pseudogene paralogs.
(0.22 MB TIF)

Figure S3 Plot of distance difference between the DBTSS and RefSeq transcription start site in scale of 10bp (left) and 300bp (right).
(0.15 MB TIF)
