# Supplementary material for: A Structural Split in the Human Genome
Source: PLoS One. 2007 Jul 11;2(7):e603. doi: 10.1371/journal.pone.0000603 (PMC1904255; doi:10.1371/journal.pone.0000603)
Supplement: Supplement S1 — Supplement 1. (0.03 MB DOC) [file pone.0000603.s001.doc]

**Supplement 1**

**Divergent promoters**

We identified 1934 pairs of human RefSeq genes adjacent to each other in head-to-head arrangement based on the annotation of the UCSC database. We categorized these pairs into three groups according to the distance between transcription start sites:

(1) < 0.3kb, or overlapping

(2) 0.3 to 1kb

(3) 1-10kb.

For genes with multiple head-to-head gene neighbours, we classified them into the group with the lowest distance between the respective transcriptional start sites (TSS; **Supplementary Table 1**).

Divergent promoters have been suggested to have a functional significance in transcription regulation, perhaps involving coordinated expression of the two relevant genes [1]. Despite the controversy of the preferential association with CpG islands [1-3], their abundance in housekeeping genes could have an as-yet-undetermined biological significance. To explore this possibility, we analyzed the abundance of CpG islands in all three types of divergent promoters. As shown in **Supplementary Table 2**, CpG islands were found to be overrepresented in comparison with all genes, particularly those less than 1 kb in length. Using Takai and Jones' (2004) statistical estimation, the expected gene proportion with PCIs = 1- (1-0.682)^2 = 89.89%, which is still lower than our observed proportion (>96% for genes with <1kb apart). This confirms the previous reports of highly abundant CpG islands in bidirectional promoters [1, 2], supporting the potential role of (de)methylation-related transcription mechanisms (i.e., either repression or activation) in coordinating gene expression.

We then compared the three groups of divergent promoters based on the eight variables, as for the PCI+/- genes. However, these groups were shown to be structurally indistinguishable in terms of intron number, evolutionary rate and expression level: genes with divergent promoters, whether < 1kb or 10 kb apart, have short introns and high expression breadth. One explanation for this may be the dominance of shorter, more broadly expressed PCI+ genes, which contribute to over 96% for two of three groups**.** Although these two characteristics seemed similar to previous reports of overrepresented housekeeping gene features, we did not observe enrichment of housekeeping genes compared to pseudogene paralogs (not shown).

Though Kanado (2005) believed that the origin of bidirectional promoters should be no later than mammalian divergence, we could not observe any significant differential evolutionary rate, quantified by Ka and Ks, between divergent and unidirectional promoters, giving no evidence of their origin being related to DNA methylation.

**References**

1. Triklein N.D, et. al, (2004). An abundance of bidirectional promoters in the human genome. *Genome Res,* **14**:62-66.

2. Adachi N. and Lieber M.R. (2002). Bidirectional Gene Organization: A Common Architectural Feature of the Human Genome. *Cell* **109**:807-809.

3. Takai D. and Jones P.A., (2004). Origins of Bidirectional Promoters: Computational Analyses of Intergenic Distance in the Human Genome. *Mol. Biol. Evol*. **21(3)**:463–467.

4. Koyanagi K.O. et.al (2005). Comparative genomics of bidirectional gene pairs and its implications for the evolution of a transcriptional regulation system. *Gene*, **353**:169-176.

5. Yamashita R. (2006) DBTSS: DataBase of Human Transcription Start Sites, progress report 2006. *Nucleic Acids Res.* **34(Database issue)**:D86-9.
