# Supplementary material for: A Structural Split in the Human Genome
Source: PLoS One. 2007 Jul 11;2(7):e603. doi: 10.1371/journal.pone.0000603 (PMC1904255; doi:10.1371/journal.pone.0000603)
Supplement: Supplement S2 — Supplement 2. (0.03 MB DOC) [file pone.0000603.s002.doc]

**Supplement 2**

**Pseudogene paralogs and housekeeping genes**

To ensure a clear classification of housekeeping genes and processed pseudogene paralogs, we examined the expression profile in terms of SAGE count and expression breadth. As shown in **S2 Figure 1**, processed pseudogenes are characterized by significantly lower expression breadth and level, whereas housekeeping genes are highly and broadly expressed, consistent with their readily distinguishable functions.
