# Supplementary material for: A Structural Split in the Human Genome
Source: PLoS One. 2007 Jul 11;2(7):e603. doi: 10.1371/journal.pone.0000603 (PMC1904255; doi:10.1371/journal.pone.0000603)
Supplement: Supplement S3 — Supplement 3. (0.03 MB DOC) [file pone.0000603.s003.doc]

**Supplement 3**

**Comparison of transcription start sites (TSS) in RefSeq and DBTSS**

DBTSS database (http://dbtss.hgc.jp) records the experimentally determined transcription start site by human cDNA clones (Yamashita, 2006). Human TSS annotation corresponding to hg17 was mapped to RefSeq annotation in UCSC. Direct comparison indicated that 82% of the genes differed by 0-10 bp in 5’ end (**S3 Figure 1**), whereas only 7% of genes with 5’ region differed by more than 100 bp, of which 76 and 62 fell within low GC and high GC group, contributing to 6% and 7% of each group respectively.

For our definition of promoter CpG islands, we considered overlapping with 2 kb upstream and 500 bp downstream of the transcription start site (TSS). Hence, we conclude that the discrepancy between RefSeq and experimentally-determined TSS introduces minimal bias into the definition of PCI-associated genes.

**References**

1. Yamashita R. (2006) DBTSS: DataBase of Human Transcription Start Sites, progress report 2006. *Nucleic Acids Res.* **34(Database issue)**:D86-9.
