# Supplementary material for: A Structural Split in the Human Genome
Source: PLoS One. 2007 Jul 11;2(7):e603. doi: 10.1371/journal.pone.0000603 (PMC1904255; doi:10.1371/journal.pone.0000603)
Supplement: Table S1 — Distribution of genes with divergent promoters, as characterised by the distance between transcriptional start sites. (0.02 MB DOC) [file pone.0000603.s004.doc]

| **Distance between TSS** | **Number of genes** |
| --- | --- |
| Overlapping | 58 (3.00%) |
| < 0.3kb | 545 (28.18%) |
| 0.3 – 1kb | 373 (19.29%) |
| 1 – 10kb | 958 (49.53%) |
| **Total** | **1934** |
