# Supplementary material for: A Structural Split in the Human Genome
Source: PLoS One. 2007 Jul 11;2(7):e603. doi: 10.1371/journal.pone.0000603 (PMC1904255; doi:10.1371/journal.pone.0000603)
Supplement: Table S2 — Proportion of divergent promoters with CpG islands among genes with different distances between transcription start sites vs. all genes. (0.02 MB DOC) [file pone.0000603.s005.doc]

|  | **<0.3kb and overlapping** | **0.3-1kb** | **1-10kb** | **All genes** |
| --- | --- | --- | --- | --- |
| PCI+ | 927 | 513 | 954 | 16655 |
| Total | 965 | 528 | 1299 | 24484 |
| Proportion (%) | **96.06** | **97.16** | 73.44 | 68.02 |
