# Supplementary figures and images for: A Structural Split in the Human Genome
Source: PLoS One. 2007 Jul 11;2(7):e603. doi: 10.1371/journal.pone.0000603 (PMC1904255; doi:10.1371/journal.pone.0000603)

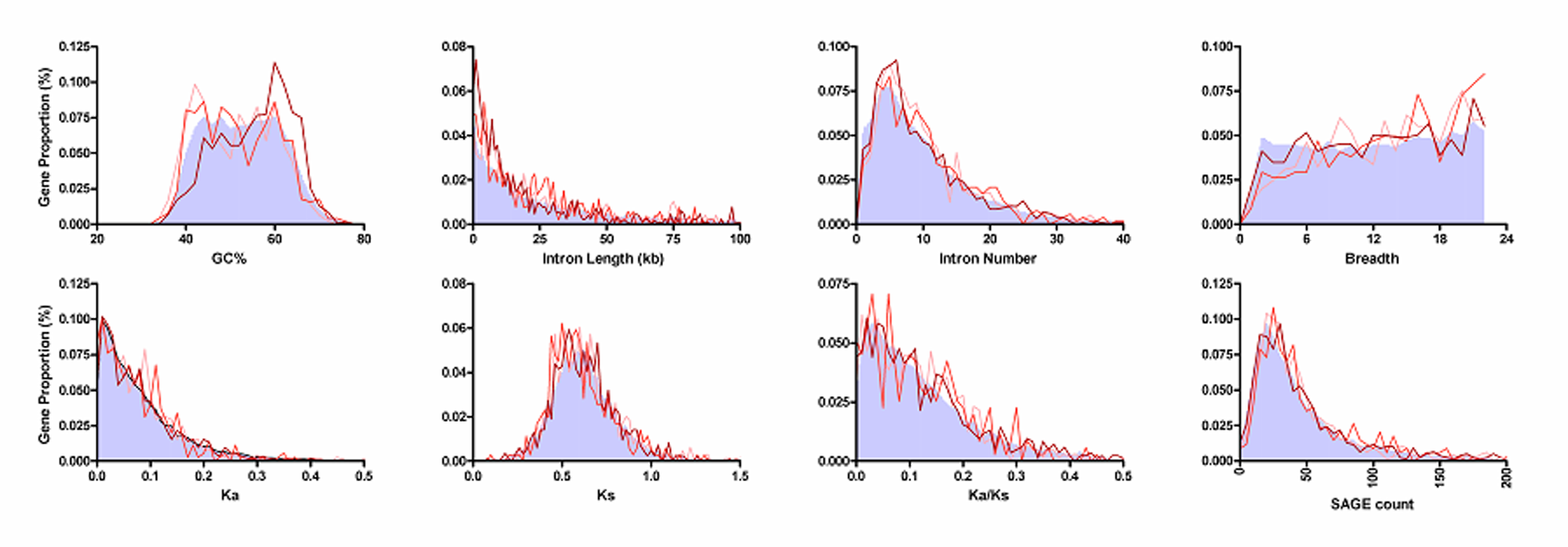

Supplement: Figure S1 — Structural characterization (Upper panel : GC content, total intron length and number of intron(s), expression breath; Lower panel : Ka, Ks, Ka/Ks and expression level) of three groups of divergent promoters, overlapping or <0.3kb (pink); 0.3-1kb (red); 1-10kb (dark red) against all genes (light blue; shaded). (0.85 MB TIF) [file pone.0000603.s006.tif]

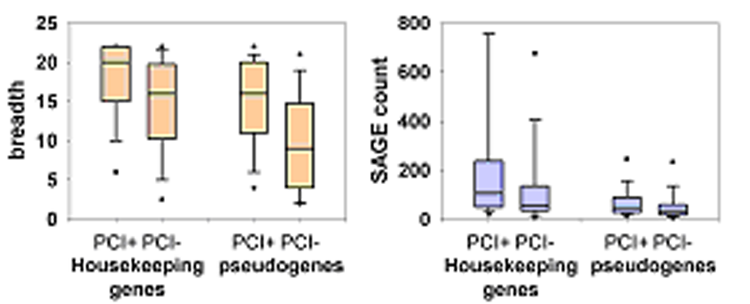

Supplement: Figure S2 — Median and quartile expression level and breadth of housekeeping genes and pseudogene paralogs. (0.22 MB TIF) [file pone.0000603.s007.tif]

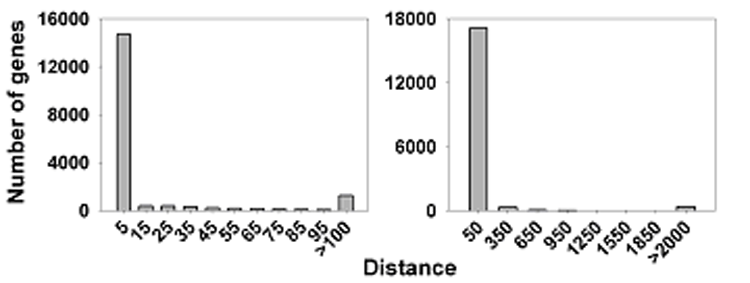

Supplement: Figure S3 — Plot of distance difference between the DBTSS and RefSeq transcription start site in scale of 10bp (left) and 300bp (right). (0.15 MB TIF) [file pone.0000603.s008.tif]
